# PatientsLikeMe and Online Patient Support Communities in Dermatology

**DOI:** 10.2196/50453

**Published:** 2024-06-26

**Authors:** Mindy D Szeto, Michelle Hook Sobotka, Emily Woolhiser, Pritika Parmar, Jieying Wu, Lina Alhanshali, Robert P Dellavalle

**Affiliations:** 1 Department of Dermatology University of Minnesota Medical School Minneapolis, MN United States; 2 Arizona College of Osteopathic Medicine Midwestern University Glendale, AZ United States; 3 College of Osteopathic Medicine Kansas City University Kansas City, MO United States; 4 Department of Dermatology University of Colorado Anschutz Medical Campus Aurora, CO United States; 5 School of Medicine Rocky Vista University College of Osteopathic Medicine Parker, CO United States; 6 Department of Dermatology SUNY Downstate College of Medicine Brooklyn New York, NY United States; 7 Dermatology National Executive Program Director Dermatology Service Minneapolis VA Medical Center Minneapolis, MN United States

**Keywords:** PatientsLikeMe, PLM, online support communities, social media, forums, discussion boards, internet, misinformation, community engagement, representation, demographics, lived experience, atopic dermatitis, prevalence

## Abstract

Online patient-oriented platforms such as PatientsLikeMe (PLM) offer a venue for individuals with various diagnoses to share experiences and build community, though they may not be representative of the larger patient population. This potentially limits generalizability and raises concerns about the spread of misinformation, emphasizing the need for informed use and health care provider engagement.

Receiving a diagnosis can transform a patient’s lifestyle, quality of life, and even their identity. Online patient-oriented platforms, such as PatientsLikeMe (PLM), can provide a medium for patients to interact with those who have similar diagnoses. PLM launched in 2005 and was originally focused on patients with amyotrophic lateral sclerosis (ALS); it has since expanded to over 850,000 members with more than 2800 health conditions, and has been featured in over 100 peer-reviewed studies [1].

PLM remains popular among patients who wish to share personal stories about their individual experiences and treatments in order to connect and learn from each other about symptom timing and onset, severity and resolution, medication effectiveness, side effects, and adherence [1]. A 2018 retrospective study assessed characteristics of PLM users with atopic dermatitis (AD) [2]. As of April 2018, 410 PLM users reported having AD; 90.45% were diagnosed by a medical professional, while 9.55% were self-diagnosed. AD was the primary condition in 61.46% of users; 32.01% of AD PLM users were in the 30-39-year age group and more were women (61%). Common symptoms reported included stress, fatigue, pain, anxious mood, and depressed mood at different levels of patient-defined severity. Users discussed experiences with successful management and nonpharmacological interventions, ranging from modafinil for insomnia to music therapy for anxiety.

However, due to the small number of PLM users reporting AD, especially for treatment data (N=28), profiles may not be representative compared to AD as described in the scientific literature. For example, some studies report a female predominance of AD, as observed in PLM, while others find no gender association [2]. Conversely, a larger study (N=21,101) of PLM users with systemic lupus erythematosus (SLE) reported similarities in age, socioeconomic status, symptom frequency, and medication use patterns when compared to the greater population of patients with SLE in the United States [3], patterns largely concordant with claims data in other diseases [4]. However, as expected, slightly more PLM users reporting SLE were female (97%, higher than 82%-93% in real-world samples) and White (68%, compared to 22%-63% in population studies) [3]. Discrepancies when comparing demographics of disease prevalence may be rooted in the self-selected nature of PLM use, where users predominantly identified as female and non-Hispanic White, and were generally younger and more highly educated than even those of other online platforms [5]. Women are also more likely to use internet sources for health information compared to men [3]. Internet experience and higher incomes have additionally been associated with the use of online tools, which may be reflective of social determinants that affect other aspects of health care delivery. Attempts to expand the accessibility and benefits of PLM to a wider audience may be worthwhile, as it has been highly valuable in promoting connections among patients, where hearing from those with similar symptoms aided others in comparable situations and forged strong relationships based on shared lived experiences and exchange of knowledge [6].

To help understand and expand the benefits of PLM to a wider audience, a cross-sectional retrospective survey in 2016-2017 investigated the potential of a customized condition-specific versus generalized PLM platform and examined the impact of community-focused upgrades sponsored by pharmaceutical partnerships [7]. A total of 377,625 PLM members were invited to take the survey with 7434 completions (5344 with community upgrades, 2045 without). The generalized platform was observed to improve knowledge, symptom management, and patient activation, with further increases in knowledge for those with upgrades. However, results were potentially biased due to respondent selection and demographics, varying levels of use, and the cross-sectional study design [7].

Despite efforts to improve utility and knowledge, PLM comes with challenges. While it can encourage patient advocacy and data-driven discussions [8], it may not be representative or generalizable to all patients, as previously mentioned, and those willing to share their experiences may already be more active and engaged in their health, with better health care access [4]. There is also a risk of spreading misinformation, as content is not reviewed by medical professionals, which may be particularly dangerous for patients with complex comorbidities (which are underreported on PLM) [3]. PLM information regarding standard of care or interventions to avoid is also lacking [2]. Encouraging health care provider acknowledgment of and engagement on these forums with patients can play an important role in promoting community-building and health literacy and developing trust and rapport while cautioning users on the potential for misinformation. While extremely beneficial to many, online platforms like PLM should not be all-encompassing resources, and informed use is paramount.

## Abbreviations

AD: atopic dermatitis
ALS: amyotrophic lateral sclerosis
PLM: PatientsLikeMe
SLE: systemic lupus erythematosus
